# An ozonesonde evaluation of spaceborne observations in the Andean tropics

**DOI:** 10.1038/s41598-022-20303-7

**Published:** 2022-09-24

**Authors:** María Cazorla, Edgar Herrera

**Affiliations:** Universidad San Francisco de Quito USFQ, Colegio de Ciencias e Ingenierías, Instituto de Investigaciones Atmosféricas, Quito, Ecuador

**Keywords:** Atmospheric chemistry, Climate sciences

## Abstract

Satellite observations of ozone in the tropics have feedback from in situ measurements at sea level stations, but the tropical Andes is a region that is yet to be included in systematic validations. In this work, ozonesondes launched from the equatorial Andes were used to evaluate total column ozone (TCO) measured by spaceborne sensors TROPOMI/S5P (2018–2021), GOME-2/MetOp-B, OMI/Aura, and OMPS/Suomi NPP (2014–2021). Likewise, we evaluated tropospheric column ozone (TrCO) measured by the first two. Additionally, we evaluated TCO and TrCO from reanalysis products MERRA-2 and CAMS-EAC4. Results indicate that TCO observations by OMPS/Suomi NPP produce the closest comparison to ozonesondes (− 0.2% mean difference) followed by OMI/Aura (+ 1.2% mean difference). Thus, they outperform the sensor with the highest spatial resolution of current satellite measurements, namely TROPOMI/S5P (+ 3.7% mean difference). This overprediction is similar to the one encountered for GOME-2/MetOp-B (+ 3.2% mean difference). A positive bias with respect to soundings was also identified in TrCO measured by TROPOMI/S5P (+ 32.5% mean difference). It was found that the climatology used by TROPOMI overpredicts ozone in the troposphere when compared with the mean of Andes measurements, while both data sets are essentially the same in the stratosphere. Regarding reanalysis products, MERRA-2 compares better to ozonesondes than CAMS, both for TCO and TrCO (mean differences are 1.9% vs. 3.3%, and 11.5% vs. 22.9%, respectively). Identifying spaceborne ozone measurements that currently perform the best over the region is relevant given the present conditions of rapidly changing atmospheric composition. At the same time, ozonesonde data in this work offer an opportunity to improve satellite observations in the Andean tropics, a challenging region for space measurements.

## Introduction

In the tropics, there have been dedicated validations of satellite products for ozone due to its major relevance to climate and the wellbeing of the global environment. Most of the tropical stations that have been part of these efforts are located at or near the sea level^1^. Such studies have been focused on the tropical troposphere^2^, where ozone is a short-lived climate forcer; the tropical stratosphere^3^, where ozone is produced and redistributed to the globe^4^; and the total column^5,6^. In contrast, the tropical Andes is a region where satellite validations have not received sufficient attention despite a daunting altitudinal gradient that challenges measurements from space. For example, some ready-to-use satellite gridded products of total column ozone (TCO) are usually smoothed spatially and temporally over cloud-free scenes and in windows of up to 3 days^7^. However, spatial smoothing of TCO over the complex Andean topography can bias measurements if highlands data become averaged with Amazon or coast side data due to differences in the depth of the atmospheric column. On the other hand, cloudiness also affects measurements from space. In the tropical Andes, cloud formation is associated to the ITCZ and the orographic uplifting factor^8^. In the case of tropospheric column ozone (TrCO), measurements from space are generally difficult because 90% of the signal is in the stratosphere^9^. Regardless, satellite algorithms rely on ozone measurements above convective clouds to estimate TrCO^10,11^. Thus, comparisons are needed to determine which of the several products available at the user-level suit best a region where validations are lacking. Therefore, the purpose of this study is to evaluate satellite and reanalysis products (TCO and TrCO) and identify data sets that compare the best to a unique set of ozone profiles collected in the equatorial Andes.

In situ sampling of ozone on board of high-altitude balloons began actively in 2014 at Universidad San Francisco de Quito’s Atmospheric Measurement Station (EMA, Spanish acronym) in Ecuador^12,13^. This effort continued into 2021 with the support of the Vienna Convention Trust Fund (VCTF). Thus, a total of 69 profiles are presented in this study. Currently, the Ecuadorian record is the only source of in situ data for satellite comparisons in the region. Available since 2018, the TROPOspheric Monitoring Instrument (TROPOMI), on board of the Sentinel-5 Precursor (S5P) satellite, has been praised for its spatial resolution and the accuracy of its retrievals^14^. Thus, we compare TCO and TrCO against ozonesondes for 2018–2021. Prior to TROPOMI, the Global Ozone Monitoring Experiment 2 (GOME-2/MetOp-B) has provided both products since 2012^15,16^. Hence, comparisons are presented for 2014–2021. For the same period, we also present comparisons of TCO from the Ozone Monitoring Instrument (OMI/Aura), and the Ozone Mapping and Profiler Suite (OMPS/Suomi NPP).

In addition to satellite observations, reanalysis products have critical relevance to understanding changes in atmospheric composition, which is particularly useful in regions where a network of coordinated observations is scarce. Thus, we perform comparisons using two relevant products: the Modern Era Retrospective analysis for Research and Applications (MERRA-2) and the Copernicus Atmospheric Monitoring Service global reanalysis (CAMS-EAC4). For these data sets, we compare TCO and TrCO against ozonesondes for the 2014–2021 period.

## Results and discussion

### Total column ozone comparisons

**Figure 1:**
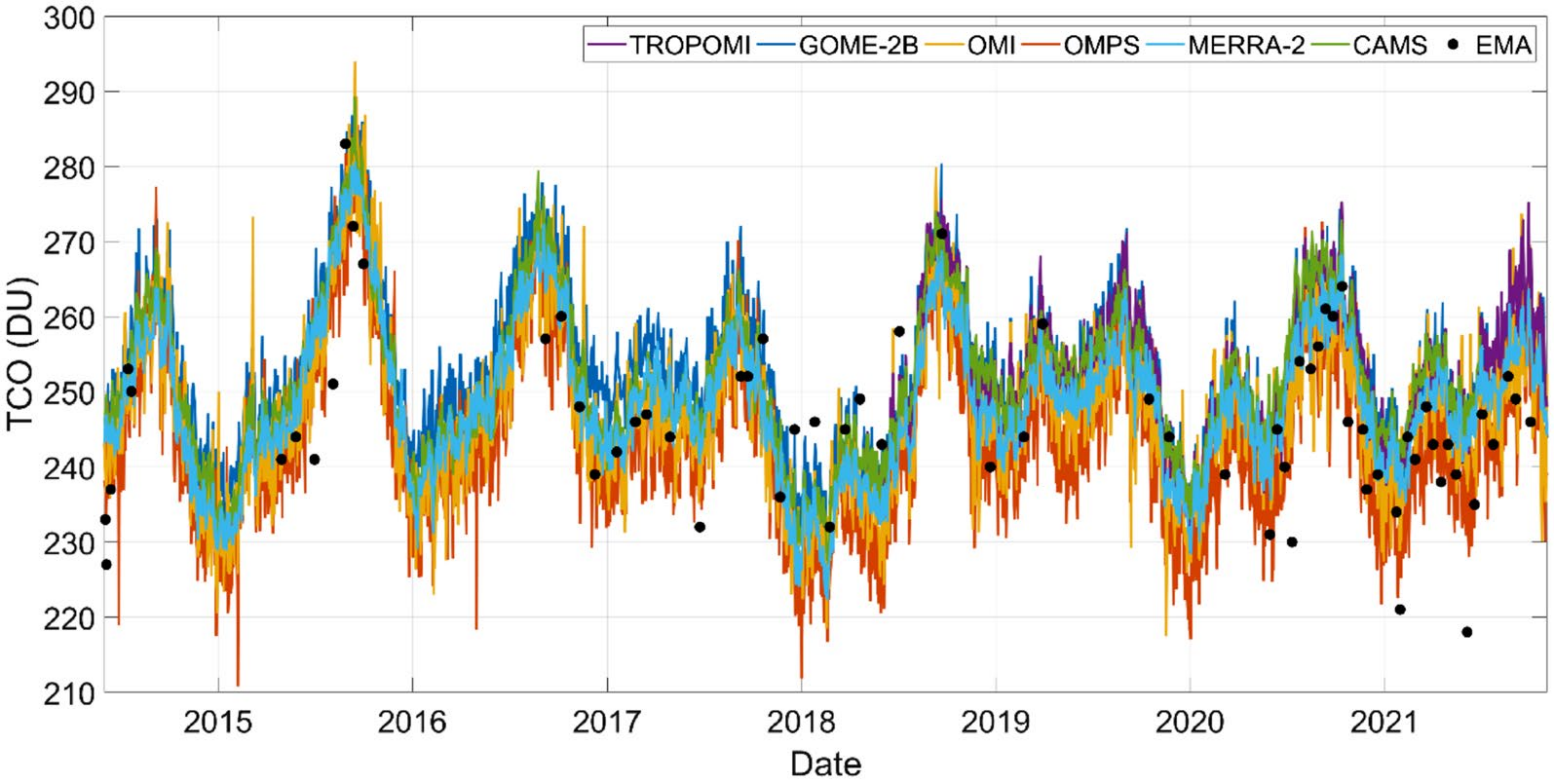
Total column ozone (TCO) time series. Measurements from Quito ozone soundings (EMA USFQ) are depicted by black dots. Satellite observations from TROPOMI/S5P, GOME-2/METOP-B, OMI/Aura, OMPS/Suomi NPP, as well as reanalysis products MERRA-2 and CAMS, are daily data and are depicted with colored lines (see figure legend).

A time series of satellite and reanalysis data alongside individual measurements at EMA in Quito (see “Methods” section) is depicted in Fig. 1. Major features observable in the time series are consistent across data sets, mainly with respect to the shape of annual profiles and the time of the year when TCO reaches a maximum (mid-September, Fig. S1). However, there are differences in the magnitude of TCO with respect to ozonesondes and among data sets, that were quantified and are discussed below. For reference, spatial and temporal resolution of spaceborne measurements, as well as the number of points compared against ozonesondes, are summarized in Table 1.

**Table 1:** Spatial and temporal resolution of satellite and reanalysis products (see “Methods” section) along with number of ozonesondes launched from EMA USFQ in Quito, Ecuador for comparisons.

Instrument/platform	Observation	Resolution		Comparison time period		
		Spatial	Temporal	Start date	End date	# Sondes
TROPOMI/S5P	TCO	7 × 3.5 km	1-day	30 April 2018	1 October 2021	39
		5.5 × 3.5 km				
	TrCO	0.5° × 1.0°		2 May 2018		
GOME-2/MetOp-B	TCO	40 × 80 km	1-day	1 June 2014	1 October 2021	69
	TrCO	1.25° × 2.5°	1-month	June 2014	October 2020	
OMI/Aura	TCO	13 × 24 km	1-day	1 June 2014	1 October 2021	69
OMPS/Suomi NPP	TCO	50 × 50 km	1-day	1June 2014	1 October 2021	69
MERRA-2	TCO	0.5° × 0.625°	1-h	1 June 2014	1 October 2021	69
	Profiles		3-h			
CAMS	TCO and profiles	0.75° × 0.75°	3-h	1 June 2014	29 June 2021	64

**Figure 2:**
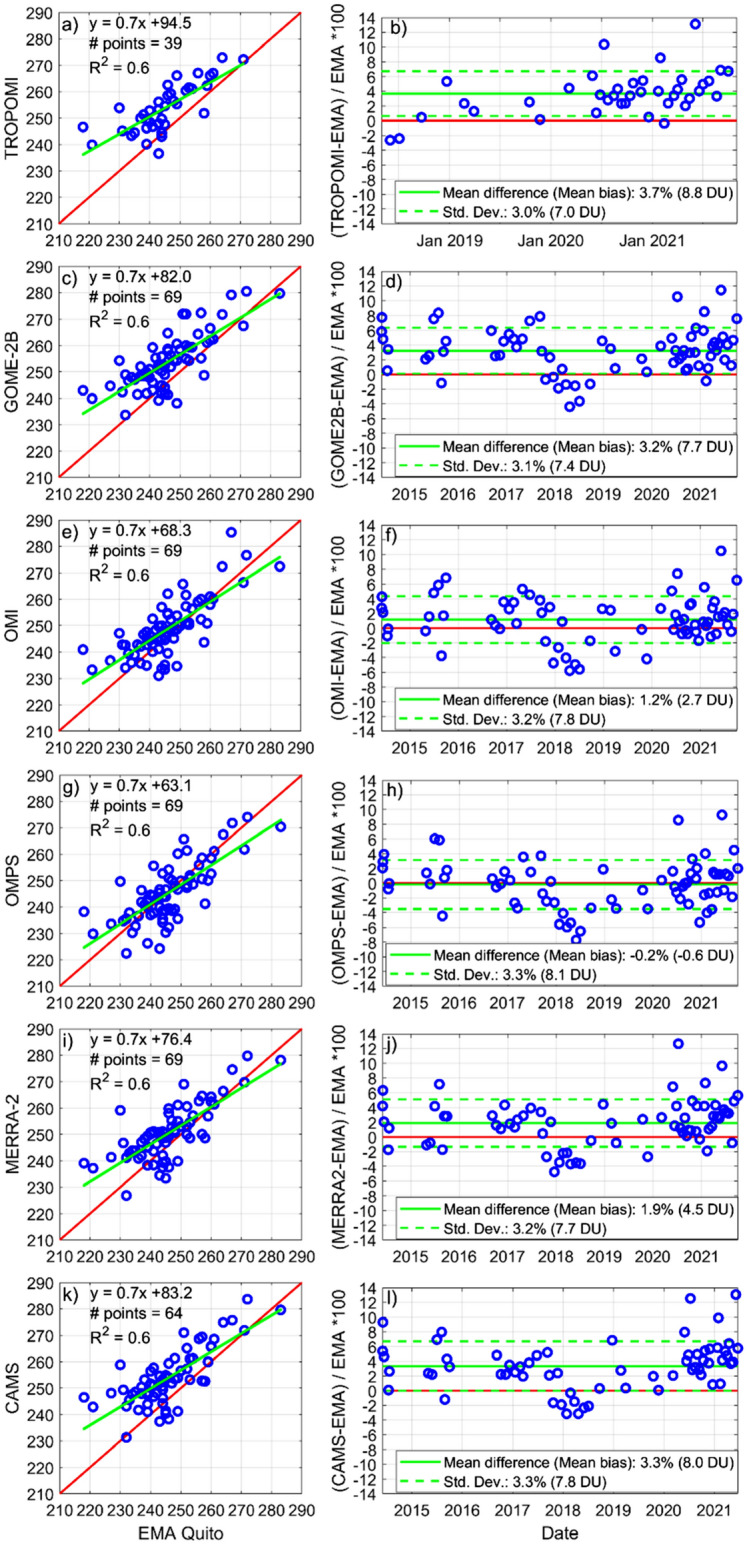
Total column ozone (TCO) comparisons between spaceborne observations and ozonesonde measurements taken in Quito, Ecuador (EMA USFQ). 1:1 comparisons and linear regressions are depicted in left panels, while mean percentage differences (or mean biases) are quantified in right panels.

TCO from TROPOMI/S5P overestimates sounding measurements with an average positive bias of + 8.8 DU, which corresponds to a mean difference of 3.7% (Fig. 2a,b). Even though the spatial resolution of TROPOMI/S5P is far superior to GOME-2/MetOP-B, the latter performs similarly as its mean bias (Fig. 2c,d) with respect to soundings is + 7.7 DU (3.2% difference). Offsets of like magnitudes in measurements by these sensors have been documented at other tropical stations^17^. In contrast, the difference between OMI/Aura and soundings (+ 2.7 DU or 1.2% mean difference; Fig. 2e,f), is a third of the one encountered for TROPOMI/5SP and GOME-2/MetOP-B. Meanwhile, TCO from OMPS/Suomi NPP was found to perform the best out of all products (Fig. 2g,h) as the mean bias with respect to soundings is − 0.6 DU (− 0.2% mean difference). In all cases, the slope of the linear regression is 0.7 and the correlation coefficient (R^2^) is 0.6. From an observational perspective, TCO from OMPS/Suomi NPP and OMI/Aura outperform TROPOMI/S5P and GOME-2/MetOP-B in the tropical Andes. Previous work shows that OMI/Aura and OMPS/Suomi agree within 2% with measurements at the majority of other tropical stations^1^.

As presented, the product with the finest spatial resolution (TROPOMI/S5P) does not yield the best comparison. This is counterintuitive as finer resolution would seem more likely to resolve the significant altitudinal gradient at the study site. However, an important aspect to consider is the structure and magnitude of ozone in the troposphere, which has been identified as a factor that causes biases in TCO retrievals^18^. Satellite algorithms use “a priori” information of ozone profiles to retrieve TCO from measurements of backscattered UV radiation^19^. Due to the high longitudinal variability of TrCO, an improvement in the TROPOMI/S5P algorithm incorporates the climatology by Ziemke et al.^20^ to provide better adjustment in the troposphere^11^. However, ozone climatologies use profiles from sites mostly at the sea level in the Atlantic and Pacific basins, while profiles at high altitude in the tropical Andes deviate from those locations mainly in the troposphere. Thus, previous research demonstrates significant differences in profile structure and in the magnitude of TrCO (lower) from stations in Galapagos (Ecuador) and Natal (Brazil), while stratospheric ozone is similar and consistent with Microwave Limb Sounder (MLS/Aura) measurements^12,13^. Hence, we compared mean differences in TCO, TrCO, and stratospheric column ozone (SCO) between Ziemke’s climatology and the compendium of EMA’s observations in Quito (Table S1). While the SCO is practically the same (+ 1.3 DU or 0.6% difference), the TCO from the climatology shows a mean bias of + 9.3 DU (3.8% mean difference), which comes from overpredicting ozone in the troposphere (+ 8 DU or 42.5%). This result is similar to the comparison of TROPOMI/S5P individual measurements against ozonesondes, discussed before. Although additional research is needed and more data are necessary, our findings remark the need to incorporate profiles in the Andean tropics to satellite climatologies, particularly to better represent TrCO in the region, which would have an overall beneficial effect in TCO.

Regarding reanalysis products, MERRA-2 yields a closer comparison (Fig. 2i,j) to observations than CAMS (Fig. 2k,l), as the mean bias is + 4.5 DU as opposed to + 8 DU (less than 2% versus 3.3% mean difference). Data assimilation in MERRA-2 incorporates TCO from OMI/Aura and stratospheric mixing ratios from MLS/Aura^21^. Meanwhile, CAMS adds to the former ozone sources TCO from GOME-2/MetOP-B^22^. From an observational perspective, MERRA-2 outperforms CAMS at estimating TCO, but additional research should be conducted to better pinpoint specific differences between both models and the data sources they use.

Finally, we quantified the differences of all products with respect to OMPS/Suomi NPP for being the data set that compares the best to in situ observations. As indicated in Fig. S2, the mean differences encountered for TROPOMI/S5P, GOME-2/MetOP-B, and OMI/Aura are 3.8%, 3.5%, and 1.4%, respectively. Regarding reanalysis, mean differences encountered for MERRA-2 and CAMS are 1.1% and 3%, respectively. Therefore, OMI/Aura and MERRA-2 agree best with OMPS/Suomi NPP.

### Tropospheric ozone comparisons

**Figure 3:**
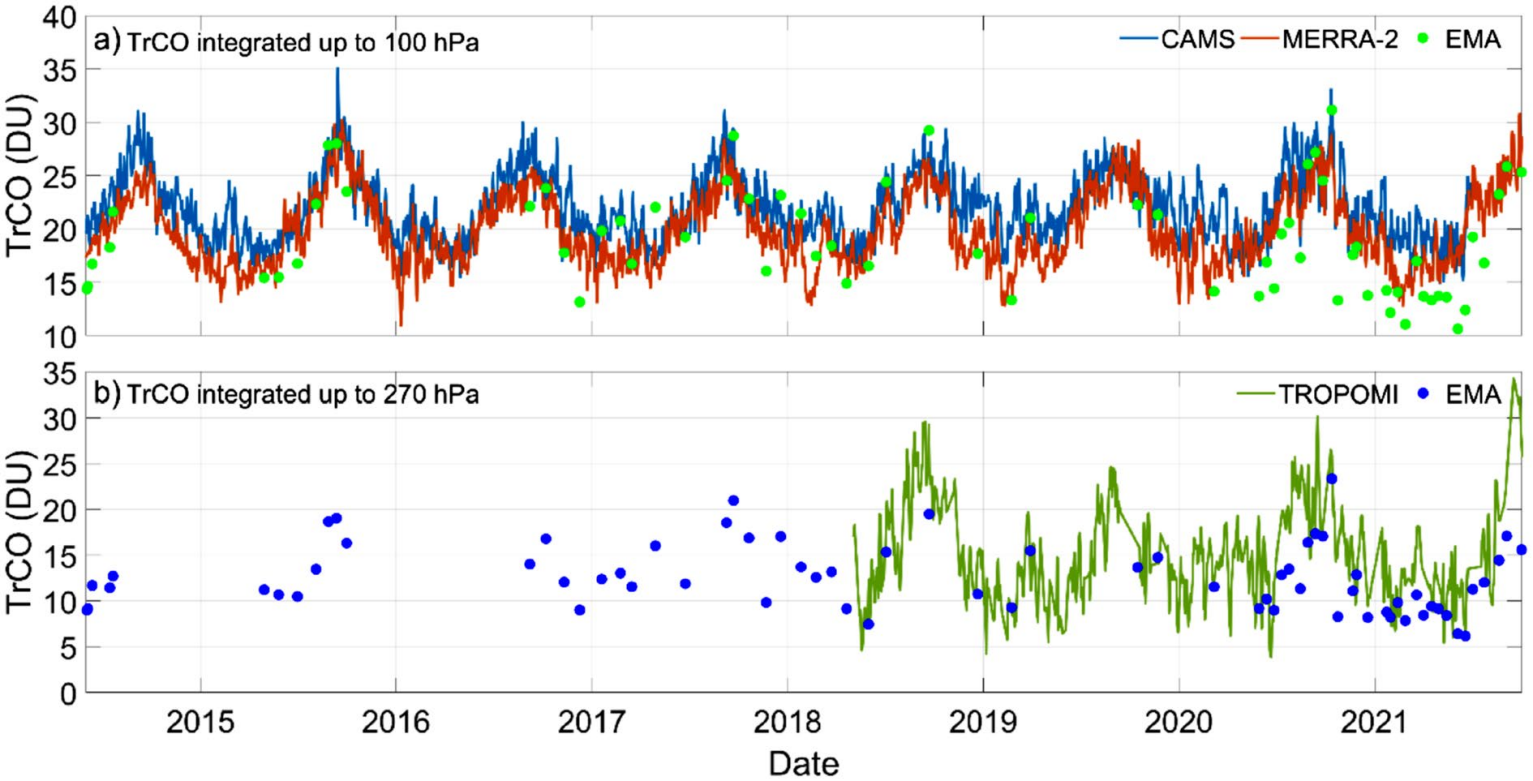
Tropospheric column ozone (TrCO) data time series. (**a**) Sounding measurements taken in Quito, Ecuador (EMA USFQ) were integrated up to 100 hPa (green dots) to compare against MERRA-2 (red line) and CAMS (blue lines). (**b**) Likewise, EMA data were integrated up to 270 hPa (blue dots) to compare against TROPOMI/S5P observations (green line).

A time series of TrCO for data sets that have daily observations is depicted in Fig. 3, although at pressure levels suitable to perform comparisons. For example, TROPOMI/S5P is available from the surface (760 hPa) up to 270 hPa, which corresponds to about 10 km of altitude. However, from high resolution profiles, the Andean tropical troposphere is located at 96 hPa (17 ± 0.7 km) (see “Methods” section, Table S2). Moreover, previous work demonstrates that the tropopause level identified with the chemical definition (“Methods” section) mostly coincides with the coldest point in the temperature profile^13^. This indicates that, unlike sites in the Pacific and Atlantic^23,24^, ozone at the Andean tropics is generally well-mixed up to tropopause level. While 270 hPa in TROPOMI/S5P is suitable to determine TrCO in the mid-latitudes, it misses the rest of the tropospheric column at the study site. We have not encountered a specific explanation on the literature on why the ready-to-use TrCO product is nominally set at 270 hPa for the entire globe^2,11^. We believe this could be somewhat misleading particularly to the end-user in the Andean tropics. Similarly, TrCO from GOME-2/MetOP-B is available up to 200 hPa, although as monthly averages (Fig. S3). In contrast, reanalysis products are available as mass mixing ratios at pressure level intervals from the surface throughout the atmospheric column. Thus, comparisons that capture the entire Andean tropical troposphere can be performed by integrating data up to 100 hPa.

**Figure 4:**
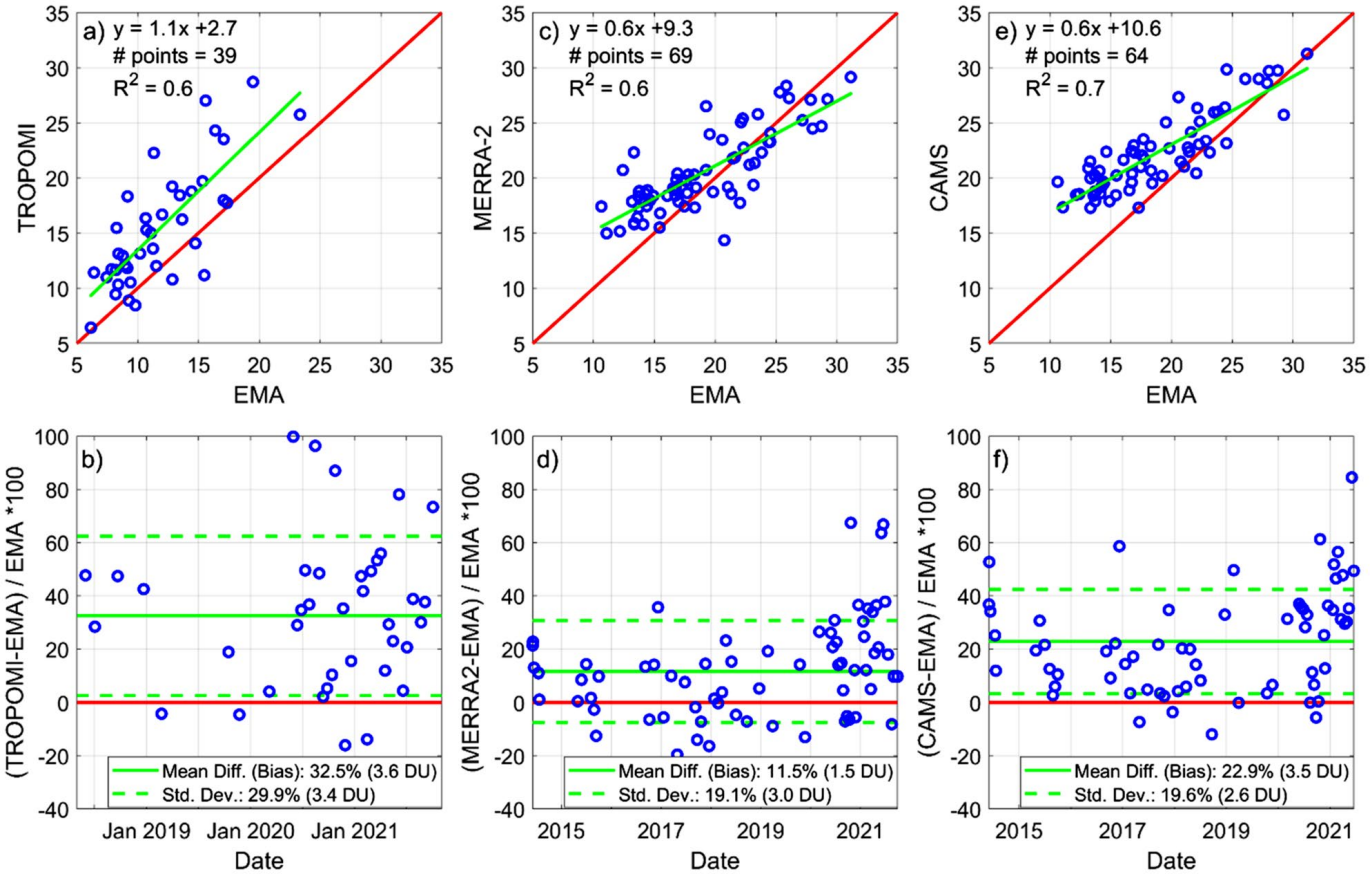
Tropospheric column ozone (TrCO) comparisons between spaceborne observations and ozonesonde measurements taken in Quito, Ecuador (EMA USFQ). 1:1 comparisons are depicted in top panels, while mean percentage differences (or mean biases) are quantified in bottom panels. Ozone soundings were integrated up to 270 hPa to compare against TROPOMI/S5P or up to 100 hPa, to compare against MERRA-2 and CAMS.

Comparing TrCO from TROPOMI/S5P against ozonesondes (integrated up to 270 hPa) yields a positive bias of + 3.6 DU or 32.5% mean difference (Fig. 4a,b). Previous research shows that tropospheric ozone in the tropical Andes is low as altitude deducts 5–7 DU from the TrCO, while ozone from the boundary layer is also low^13^. However, TROPOMI/S5P measures generally higher values even though observations correspond to a fraction of the column. In recent research that assessed the quality of TROPOMI/S5P TrCO against ozonesondes in the tropics, biases were also encountered. For example, differences at several sea level stations were found to be + 4 DU (up to 22% higher) when data were averaged over 2 years, while the overall positive bias was reported at + 2.3 DU (or 11%) when data was smoothed across longitudes^2^. The cause for this positive bias was reported not to be fully understood and was partially attributed to possible systematic differences in the time of measurements, provided TrCO follows a diurnal pattern. We also report a positive bias in the Andean tropics that also needs further investigation. Partially, this overprediction likely comes from satellite climatologies overpredicting ozone in the Andean troposphere, as discussed in the previous section. However, additional comparisons are needed with more data in the future to better understand the nature and persistence of these differences.

MERRA-2 TrCO compared against soundings up to 100 hPa yields the best comparison in the troposphere (Fig. 4c,d). The bias with respect to ozonesondes is + 1.5 DU (11.5% difference), which we consider low given the difficulty at capturing correctly TrCO at this highly complex site where validations have not been performed before. In the case of CAMS, integration was also done up to 100 hPa. The mean bias (Fig. 4e,f) is + 3.5 DU (23%), which doubles that of MERRA-2, but the correlation coefficient for the linear regression is higher (R^2^ = 0.8 vs 0.6). In contrast, TrCO from GOME-2/MetOP-B is only available to the final user as monthly averages. Thus, this product was only qualitatively compared against EMA in a time series (Fig. S3), but data are insufficient to draw quantitative conclusions.

Finally, from the perspective of the end user, currently the MERRA-2 TrCO suits best the Andean tropics. First, because data integration can be done up to a pressure level that captures the entirety of the Andean tropospheric column. Second, because differences from observations are the least.

## Conclusions

In this work we evaluate user-level satellite products at the Andean tropics against a unique set of ozonesondes launched from Quito, Ecuador between 2014 and 2021. Total column ozone from OMPS/Suomi NPP and OMI/Aura outperform TROPOMI/S5P and GOME-2/MetOP-B. Thus, mean differences are − 0.2% and 1.2% versus 3.7% and 3.2%, respectively. Differences with TROPOMI/S5P, which has the highest spatial resolution, are unexpected and require more research. A comparison of Andean profiles against the climatology used by TROPOMI/S5P to adjust ozone in the troposphere indicates that the latter overpredicts the tropospheric column ozone, which offsets the TCO climatology in 3.8%. Consistently, comparing TrCO from TROPOMI/S5P against ozonesondes between 2018 and 2021 yields a + 32.5% difference. Given the significant zonal variability in tropospheric ozone and the complexity of the study site, it follows that including Andean profiles in climatologies used for satellite retrievals would contribute to improve spaceborne measurements over this high altitude region particularly in the troposphere. Regarding reanalysis products, MERRA-2 yields better comparisons against in situ data than CAMS, both in the total and the tropospheric columns. Thus, mean differences between MERRA-2 and CAMS are 1.9% and 3.3% in the total column, and 11.5% and 22.9% in the troposphere, respectively. This work identifies for the first time satellite products that currently perform the best at representing total and tropospheric column ozone in the Andean tropics, a challenging region for space measurements. Such identification is important for climate studies under the current regime of rapidly changing atmospheric composition. At the same time, the data set presented in this study offers a unique opportunity to improve satellite measurements in tropical Andean South America, as it closes the observational gap that separates the Pacific from the Atlantic.

## Methods

### Ozonesonde data

Between June 2014 and October 2021, 69 ozone soundings were launched from EMA station at Universidad San Francisco de Quito in Ecuador. The station is a rooftop facility located at coordinates 0.19° S, 78.4° W, and 2414 m a.s.l. Ozone sounding protocols follow Standard operation procedures^25,26^ and are described elsewhere along with geographical features of the launching site^12,27^. Briefly, EN-SCI electrochemical concentration cells (ozonesondes) are conditioned three times prior to launch with cathode and anode solutions (1% KI and 1/10th buffer) prepared and aged on-site. Ozonesondes are coupled to Intermet Systems radiosondes for P–T–U (pressure, temperature, humidity) measurements. All readings are quality controlled before balloon launch by comparing against ground-station measurements (a Thermo 49i ozone analyzer and meteorological instrumentation). Data acquisition is done using the SkySonde software^28^.

In the time series, on 6 June 2021 there is a low ozonesonde point (visible in Fig. 1). However, metadata shows no indication of possible instrumental malfunctioning or error in ozonesonde conditioning for which analysis includes the entire data set.

Table S2 provides details of ozonesonde launch date, time, turn altitude and pressure, as well as ozone column measurements determined with the same methodology followed in previous work^12,13^. Thus, TCO was obtained by integrating ozone up to balloon burst altitude and adding the SBUV climatology extrapolation by McPeters and Labow^19^ (add-on table available at https://acd-ext.gsfc.nasa.gov/anonftp/toms/). TrCO in Table S2 was integrated from the surface up to the chemically defined tropopause (marked by the level at which ozone searched from above in the profile reaches 100 ppbv^24,29^). SCO was determined from the difference between TCO and TrCO. Ozonesode data collected at EMA can be accessed at https://observaciones-iia.usfq.edu.ec. For comparisons of TrCO, ozonesonde data were integrated up to 270 hPa to compare against TROPOMI/S5P, and up to 100 hPa to compare against MERRA-2 and CAMS.

### Satellite and reanalysis data

TROPOMI/S5P daily data were obtained from the GES DISC website (https://disc.gsfc.nasa.gov/), from April 2018 to October 2021. The TCO data set used was S5P_OFFL_L2__O3^30,31^, which was downloaded for the region bounded by longitudes [− 79°, − 78º] and latitudes [− 0.5°, 0.5°]. Invalid data were filtered out and the remaining data were averaged over the region. The TrCO data set used was S5P_OFFL_L2__O3_TCL^32,33^, which was downloaded for the grid centered at 78.5° W, 0.25° S. Likewise, flags were applied to filter out invalid data.

Overpass data for Quito for instruments GOME-2/MetOp-B, OMI/Aura, and OMPS/ Suomi NPP are available at the Aura Validation Data Center (https://avdc.gsfc.nasa.gov/pub/data/satellite/). Daily data were downloaded from June 2014 to October 2021. The data sets used were GOME-2/METOP-B L2 V03 TCO; OMI L2 TCO OMTO3^34^; and OMPS L2 NMTO3^35^. All these data sets cover a longitude range that overlaps east and west of the narrow Andes mountain-chain. Thus, data outside of the [− 79°, − 78°] region were filtered out for being observations that correspond either to the Ecuadorian Amazon or the coast side.

MERRA-2 TCO hourly gridded data, centered at 78.125° W and 0° S, were downloaded from the Giovanni online data system, developed and maintained by the NASA GES DISC (https://giovanni.gsfc.nasa.gov/giovanni/). The data set used was M2T1NXSLV_5_12_4_TO3^36^. For tropospheric ozone, gridded vertical profiles at different pressure levels (3 h data) were downloaded from the GES DISC website^37^. From this product, the mass mixing ratio and temperature at each pressure level were used to obtain the corresponding ozone number concentration (molecules cm^−3^). Consequently, the column in each layer was determined using the thickness of the layer. Finally, data were integrated up to 100 hPa to find the TrCO in DU. This procedure was followed for every profile.

CAMS global reanalysis (EAC4) products^22^ were downloaded from the Copernicus Atmosphere Data Store (ADS, https://ads.atmosphere.copernicus.eu/#!/home). TCO and ozone vertical profiles were obtained as gridded (3 h) data for four grids centered at: [78.75° W, 0.5° S], [78.75° W, 0.25° N], [78° W, 0.5° S], and [78° W, 0.25° N]. These data were averaged for comparisons. To obtain the TrCO, mass mixing ratio profiles were treated as for MERRA-2. The CAMS data set does not include the corresponding height for each pressure level. Hence, a mean pressure profile at EMA was used to find heigh levels and layer thicknesses.

To perform direct comparisons of TCO and TrCO against ozonesondes, high-density satellite or reanalysis observations were interpolated to the EMA time stamp (we tested this method by comparing against daily averages of satellite or reanalysis data and results were essentially the same). Subsequently, data sets were compared using two methods. First, 1:1 comparisons and linear regressions were determined between satellite or reanalysis products and EMA ozonesondes (the number of data pairs is presented in Table 1). Second, the mean bias and percentage difference along with the 1-sigma uncertainty (1 standard deviation) were quantified. To this end, the expression used was: (Measurement-Ozonesonde)/Ozonesonde. The average of values from the latter equation in Dobson Units (DU) is presented as the mean bias (or mean difference if the percentage is extracted) as in Hubert et al.^2^. A similar procedure was followed to compare satellite or reanalysis products to the data set that best compared to ozonesondes (OMPS/Suomi NPP for total column and MERRA-2 for tropospheric column, the number of data pairs compared are indicated in Figs. S2, S4).

## Supplementary Information

Supplementary Information.

## Data Availability

The datasets generated and/or analysed during the current study are available at: Quito soundings from EMA USFQ: https://observaciones-iia.usfq.edu.ec (\USFQ Data). Ozone column measurements are compiled in the Supplementary Information, Table S2. Satellite data: TROPOMI/S5P data are available from the NASA GES DISC website https://disc.gsfc.nasa.gov/. Overpass data from GOME-2/MetOp-B, OMI/Aura, and OMPS/ Suomi NPP are available at the Aura Validation Data Center: (https://avdc.gsfc.nasa.gov/pub/data/satellite/). Reanalysis data: MERRA-2 data are available from the Giovanni online data system, developed and maintained by the NASA GES DISC (https://giovanni.gsfc.nasa.gov/giovanni/). CAMS global reanalysis (EAC4) products are available at the Copernicus Atmosphere Data Store (ADS) (https://ads.atmosphere.copernicus.eu/#!/home). Specific products used from the above sources are described and referenced in “Methods” section.
