# Supplementary material for: An ozonesonde evaluation of spaceborne observations in the Andean tropics
Source: Sci Rep. 2022 Sep 24;12:15942. doi: 10.1038/s41598-022-20303-7 (PMC9509352; doi:10.1038/s41598-022-20303-7)
Supplement: Supplementary file 1 — Supplementary Information. [file 41598_2022_20303_MOESM1_ESM.docx]

**Supplementary Information**

**An ozonesonde evaluation of spaceborne observations in the Andean tropics**

M. Cazorla^1*^, E. Herrera^1^

^1^Universidad San Francisco de Quito USFQ, Colegio de Ciencias e Ingenierías, Instituto de Investigaciones Atmosféricas, Quito, Ecuador.

*mcazorla@usfq.edu.ec

**Supplementary Figures**


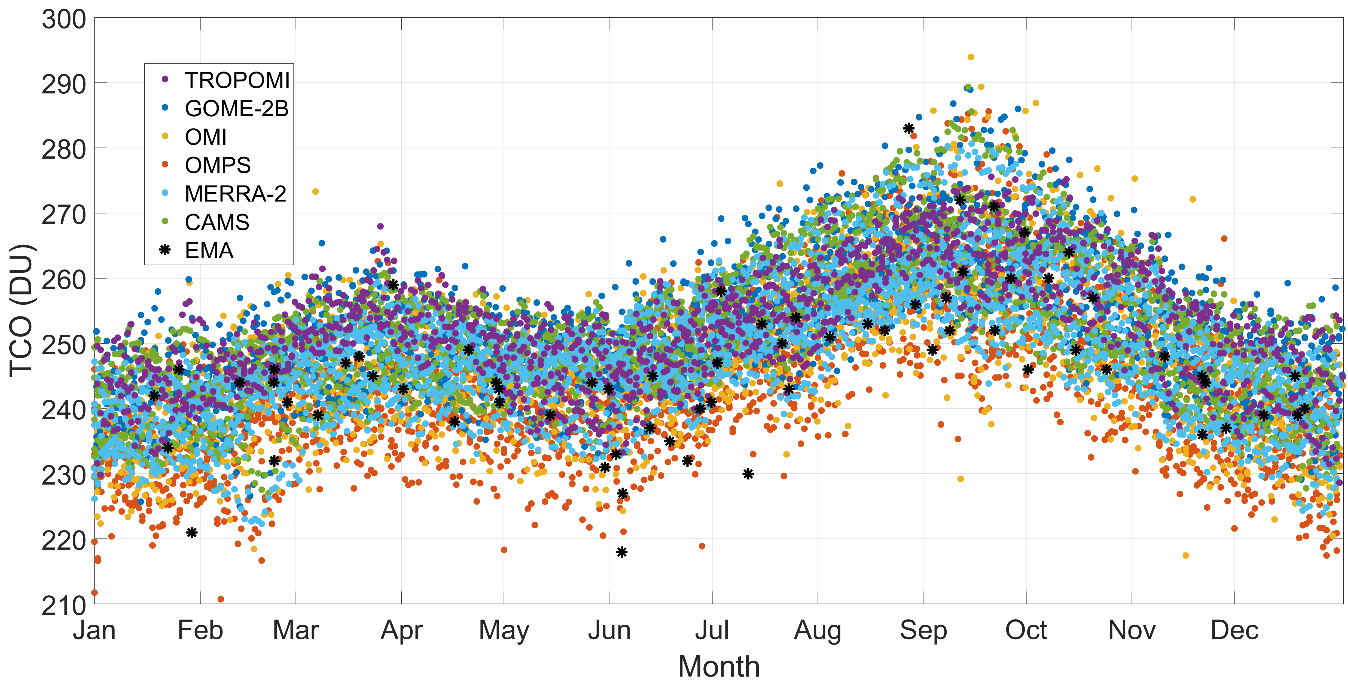


**Figure S1** TCO time series overlapped annually for Quito ozonesondes (EMA USFQ), as well as TROPOMI/S5P, GOME-2/MetOP-B, OMI/Aura, OMPS/Suomi NPP, MERRA-2 and CAMS. The March and September equinoxes^13^ mark a semiannual pattern.


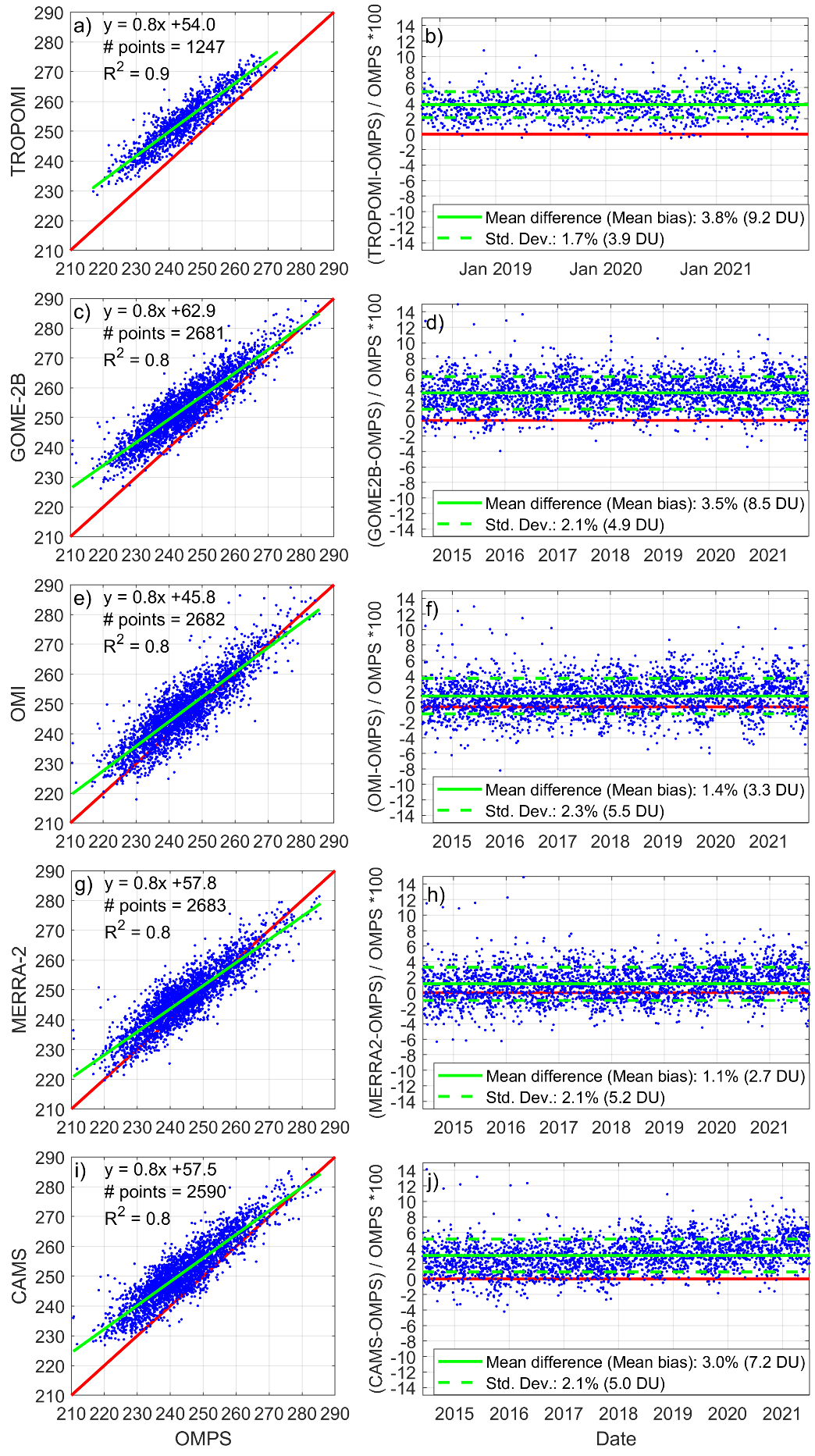


**Figure S2** Comparisons of TCO observations from TROPOMI/S5P, GOME-2/MetOP-B, OMI/Aura, MERRA-2, and CAMS against OMPS/Suomi NPP.


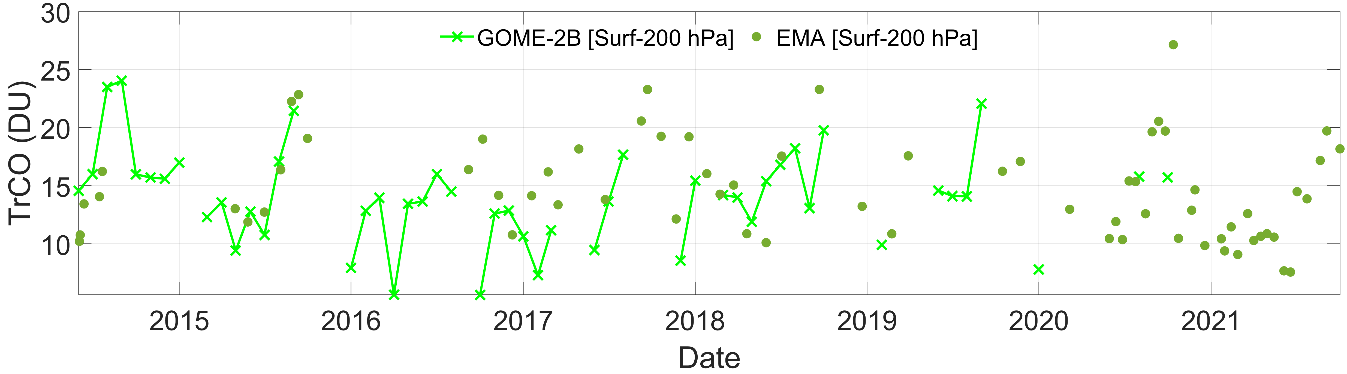


**Figure S3** TrCO time series for EMA individual soundings (integrated up to 200 hPa) and GOME-2/MetOP-B (monthly averages).

**Supplementary Tables**

**Table S1** Comparison of total, stratospheric, and tropospheric column ozone (TCO, SCO, and TrCO) between the Ziemke et. al. 2011^18^ climatology and mean data from 69 ozone soundings (Table S2) taken in Quito, Ecuador (EMA USFQ) between 2014 and 2021.

| **Data set** | **Mean TCO (DU)** | **Mean SCO (DU)** | **Mean TrCO (DU)** |
| --- | --- | --- | --- |
| Ziemke et. al. 2011^18^ | 254.7 | 228 | 26.7 |
| EMA USFQ soundings | 245.4 | 226.7 | 18.7 |
| (Ziemke-EMA)/EMA | 9.3 (3.8%) | 1.3 (0.6%) | 8.0 (42.5%) |

**Table S2** Details and measurements of Quito ozone soundings taken at EMA USFQ, which include: flight number, date and time, turn altitude and pressure, TCO, TrCO, SCO, tropopause height (chemical and thermal definitions), and cold point tropopause (CPT) temperature. TCO here presented is 2.7 DU higher, on average, than what was reported on previous work^12^ due to data reprocessing (<https://observaciones-iia.usfq.edu.ec>).

| **No.** | **Date** | **Time UTC** | **Turn Alt. (km)** | **Turn Press. (mb)** | **TCO (DU)** | **TrCO (DU)** | **SCO (DU)** | **Tropopause (km)** | | **CPT (°C)** |
| --- | --- | --- | --- | --- | --- | --- | --- | --- | --- | --- |
|  |  |  |  |  |  |  |  | **Chem.** | **CPT** |  |
| 1 | 02/06/2014 | 18:15:05 | 31.6 | 9.2 | 233 | 15.0 | 218.0 | 17.1 | 17.3 | -82.7 |
| 2 | 04/06/2014 | 17:11:35 | 31.1 | 9.9 | 227 | 14.8 | 212.2 | 16.7 | 18.0 | -81.9 |
| 3 | 12/06/2014 | 17:12:51 | 24.5 | 27.4 | 237 | 17.4 | 219.6 | 17.2 | 17.1 | -81.1 |
| 4 | 15/07/2014 | 12:54:37 | 31.4 | 9.5 | 253 | 18.6 | 234.4 | 16.9 | 16.5 | -80.0 |
| 5 | 21/07/2014 | 11:58:55 | 29.9 | 11.6 | 250 | 21.2 | 228.8 | 16.3 | 18.3 | -78.4 |
| 6 | 29/04/2015 | 17:23:50 | 31.2 | 9.8 | 241 | 16.0 | 225.0 | 17.4 | 17.0 | -87.7 |
| 7 | 26/05/2015 | 18:21:46 | 31.7 | 9.3 | 244 | 15.9 | 228.1 | 17.0 | 16.6 | -82.2 |
| 8 | 30/06/2015 | 18:41:42 | 31.7 | 9.2 | 241 | 17.6 | 223.4 | 17.4 | 16.7 | -81.7 |
| 9 | 04/08/2015 | 12:17:45 | 31.4 | 9.6 | 251 | 21.5 | 229.5 | 17.0 | 17.2 | -80.8 |
| 10 | 27/08/2015 | 14:02:34 | 31.6 | 9.4 | 283 | 28.0 | 255.0 | 16.6 | 16.3 | -79.1 |
| 11 | 11/09/2015 | 14:06:00 | 31.1 | 10.3 | 272 | 28.2 | 243.8 | 16.7 | 16.4 | -83.6 |
| 12 | 30/09/2015 | 12:44:59 | 29.6 | 12.5 | 267 | 23.6 | 243.4 | 16.6 | 16.5 | -78.2 |
| 13 | 06/09/2016 | 12:54:19 | 30.7 | 10.4 | 257 | 22.5 | 234.5 | 16.9 | 17.0 | -82.1 |
| 14 | 06/10/2016 | 12:52:31 | 30.1 | 11.8 | 260 | 24.6 | 235.4 | 17.3 | 17.1 | -82.2 |
| 15 | 09/11/2016 | 12:57:17 | 30.4 | 11.0 | 248 | 18.8 | 229.2 | 17.6 | 18.3 | -83.8 |
| 16 | 08/12/2016 | 13:03:31 | 31.8 | 8.7 | 239 | 14.1 | 224.9 | 17.7 | 16.9 | -86.5 |
| 17 | 18/01/2017 | 12:55:47 | 31.5 | 9.0 | 242 | 20.9 | 221.1 | 17.6 | 17.7 | -84.0 |
| 18 | 22/02/2017 | 15:34:29 | 32.2 | 8.2 | 246 | 21.2 | 224.9 | 16.9 | 16.7 | -81.1 |
| 19 | 15/03/2017 | 14:55:31 | 32.3 | 8.2 | 247 | 17.6 | 229.4 | 17.3 | 17.2 | -83.6 |
| 20 | 28/04/2017 | 17:56:10 | 30.9 | 10.3 | 244 | 22.8 | 221.3 | 17.2 | 17.0 | -83.4 |
| 21 | 23/06/2017 | 17:45:29 | 31.6 | 9.5 | 232 | 19.3 | 212.7 | 16.7 | 17.7 | -78.4 |
| 22 | 08/09/2017 | 14:01:15 | 29.5 | 12.7 | 252 | 24.6 | 227.4 | 16.6 | 16.3 | -81.6 |
| 23 | 21/09/2017 | 17:59:58 | 28.7 | 14.3 | 252 | 29.0 | 223.0 | 16.7 | 16.4 | -79.7 |
| 24 | 20/10/2017 | 13:04:32 | 31.7 | 9.4 | 257 | 23.3 | 233.8 | 17.1 | 16.9 | -81.5 |
| 25 | 21/11/2017 | 14:29:53 | 30.5 | 11.0 | 236 | 16.6 | 219.5 | 17.2 | 16.5 | -85.1 |
| 26 | 18/12/2017 | 17:29:20 | 31.5 | 9.6 | 245 | 24.4 | 220.7 | 17.9 | 17.8 | -87.2 |
| 27 | 25/01/2018 | 15:48:02 | 29.7 | 12.0 | 246 | 22.3 | 223.7 | 17.3 | 17.0 | -83.8 |
| 28 | 22/02/2018 | 17:12:35 | 30.5 | 10.3 | 232 | 18.1 | 213.9 | 17.1 | 17.5 | -83.3 |
| 29 | 23/03/2018 | 13:48:40 | 31.2 | 10.3 | 245 | 19.0 | 226.0 | 17.4 | N/A | N/A |
| 30 | 20/04/2018 | 14:08:00 | 30.7 | 10.6 | 249 | 15.7 | 233.3 | 17.3 | 17.1 | -84.3 |
| 31 | 31/05/2018 | 14:18:05 | 30.4 | 11.3 | 243 | 16.1 | 227.0 | 16.3 | 16.6 | -81.7 |
| 32 | 03/07/2018 | 14:57:43 | 29.6 | 12.7 | 258 | 21.7 | 236.3 | 15.2 | 17.2 | -77.5 |
| 33 | 21/09/2018 | 13:56:13 | 31.5 | 9.6 | 271 | 29.2 | 241.8 | 16.6 | 16.4 | -80.0 |
| 34 | 21/12/2018 | 14:05:40 | 30.9 | 10.2 | 240 | 18.6 | 221.4 | 17.3 | 17.4 | -85.9 |
| 35 | 22/02/2019 | 13:54:05 | 31.1 | 10.0 | 244 | 14.2 | 229.84 | 17.6 | 17.0 | -83.9 |
| 36 | 29/03/2019 | 13:30:37 | 30.9 | 10.3 | 259 | 21.6 | 237.38 | 17.2 | 16.8 | -83.3 |
| 37 | 15/10/2019 | 13:26:51 | 31.8 | 8.9 | 249 | 21.1 | 227.94 | 15.9 | 17.1 | -79.7 |
| 38 | 22/11/2019 | 13:09:04 | 26.2 | 20.4 | 244 | 21.3 | 222.66 | 16.5 | 16.4 | -81.7 |
| 39 | 06/03/2020 | 12:22:31 | 30.7 | 10.6 | 239 | 14.9 | 224.07 | 17.8 | 17.0 | -82.7 |
| 40 | 29/05/2020 | 12:41:51 | 33.4 | 7.4 | 231 | 14.5 | 216.50 | 17.5 | 17.3 | -83.2 |
| 41 | 12/06/2020 | 12:36:29 | 30.9 | 10.6 | 245 | 16.7 | 228.28 | 16.5 | 17.6 | -79.7 |
| 42 | 26/06/2020 | 12:32:46 | 30.7 | 10.9 | 240 | 15.2 | 224.78 | 17.2 | 17.3 | -81.6 |
| 43 | 10/07/2020 | 12:32:09 | 32.7 | 8.1 | 230 | 18.8 | 211.18 | 16.1 | 16.8 | -78.4 |
| 44 | 24/07/2020 | 12:35:49 | 31.8 | 9.0 | 254 | 20.2 | 233.78 | 16.4 | 16.2 | -78.8 |
| 45 | 14/08/2020 | 12:45:08 | 33.6 | 7.1 | 253 | 16.5 | 236.54 | 16.2 | 17.2 | -81.4 |
| 46 | 28/08/2020 | 12:35:27 | 30.8 | 10.5 | 256 | 24.8 | 231.22 | 15.8 | 16.2 | -78.1 |
| 47 | 11/09/2020 | 12:35:15 | 32.4 | 8.4 | 261 | 25.0 | 236.01 | 15.7 | 16.1 | -79.2 |
| 48 | 25/09/2020 | 12:43:13 | 31.4 | 9.8 | 260 | 24.8 | 235.24 | 16.8 | 16.6 | -82.4 |
| 49 | 12/10/2020 | 12:45:08 | 30.6 | 10.8 | 264 | 31.1 | 232.90 | 16.5 | 16.8 | -80.2 |
| 50 | 23/10/2020 | 12:40:25 | 29.9 | 2.0 | 246 | 14.3 | 231.69 | 17.6 | 17.4 | -87.8 |
| 51 | 20/11/2020 | 12:45:02 | 32.2 | 8.7 | 245 | 18.2 | 226.81 | 17.2 | 17.1 | -84.5 |
| 52 | 27/11/2020 | 12:51:30 | 32.6 | 8.2 | 237 | 18.7 | 218.35 | 16.9 | 17.4 | -79.9 |
| 53 | 18/12/2020 | 12:54:16 | 32.2 | 8.4 | 239 | 13.4 | 225.62 | 16.6 | 16.4 | -80.5 |
| 54 | 22/01/2021 | 12:39:18 | 32.3 | 8.1 | 234 | 15.2 | 218.85 | 17.4 | 17.7 | -85.9 |
| 55 | 29/01/2021 | 12:48:14 | 29.7 | 11.7 | 221 | 13.0 | 207.99 | 17.4 | 17.1 | -84.9 |
| 56 | 12/02/2021 | 12:54:48 | 32.9 | 7.5 | 244 | 14.5 | 229.53 | 17.0 | 18.0 | -86.5 |
| 57 | 26/02/2021 | 12:44:57 | 24.6 | 26.4 | 241 | 12.0 | 229.03 | 17.6 | 17.4 | -84.3 |
| 58 | 19/03/2021 | 12:37:43 | 31.9 | 8.7 | 248 | 17.0 | 231.03 | 16.6 | 17.4 | -82.3 |
| 59 | 01/04/2021 | 12:40:34 | 31.6 | 9.1 | 243 | 14.4 | 228.61 | 17.2 | 16.9 | -82.7 |
| 60 | 16/04/2021 | 12:47:10 | 31.7 | 9.2 | 238 | 13.9 | 224.06 | 17.6 | 17.0 | -85.8 |
| 61 | 29/04/2021 | 12:46:29 | 32.1 | 8.4 | 243 | 14.4 | 228.65 | 17.1 | 17.1 | -80.1 |
| 62 | 14/05/2021 | 12:39:13 | 31.4 | 9.5 | 239 | 14.2 | 224.79 | 17.1 | 16.5 | -81.8 |
| 63 | 04/06/2021 | 12:37:54 | 31.1 | 9.9 | 218 | 10.8 | 207.21 | 16.7 | 18.1 | -81.5 |
| 64 | 18/06/2021 | 12:44:40 | 32.0 | 8.8 | 235 | 12.5 | 222.52 | 16.7 | 16.3 | -79.0 |
| 65 | 02/07/2021 | 12:44:14 | 30.8 | 10.6 | 247 | 19.6 | 227.42 | 16.8 | 16.7 | -77.0 |
| 66 | 23/07/2021 | 12:40:00 | 31.0 | 10.4 | 243 | 17.2 | 225.78 | 17.0 | 16.6 | -83.4 |
| 67 | 20/08/2021 | 12:42:00 | 31.3 | 9.6 | 252 | 21.7 | 230.29 | 15.6 | 16.5 | -75.8 |
| 68 | 03/09/2021 | 13:02:02 | 31.5 | 9.5 | 249 | 25.8 | 223.19 | 16.5 | 16.4 | -79.5 |
| 69 | 01/10/2021 | 12:48:23 | 30.9 | 10.6 | 246 | 21.2 | 224.80 | 14.7 | 15.2 | -70.5 |
